# Comparison of the more than 5-year clinical outcomes of cervical disc arthroplasty versus anterior cervical discectomy and fusion: A protocol for a systematic review and meta-analysis of prospective randomized controlled trials

**DOI:** 10.1097/MD.0000000000005733

**Published:** 2016-12-23

**Authors:** Min-Min Shao, Chun-Hui Chen, Zhong-Ke Lin, Xiang-Yang Wang, Qi-Shan Huang, Yong-Long Chi, Ai-Min Wu

**Affiliations:** a Department of Orthopedics, Second Affiliated Hospital of Wenzhou Medical University, Second Medical College of Wenzhou Medical University; b Department of ENT and Neck Surgery, Wenzhou Center Hospital, Dingli Hospital of Wenzhou Medical University, Wenzhou, Zhejiang, People's Republic of China.

**Keywords:** anterior cervical discectomy and fusion, cervical disc arthroplasty, cervical vertebrae, meta-analysis, systematic review

## Abstract

**Background:**

Anterior cervical discectomy and fusion (ACDF) was almost the “golden standard” technique in treatment of symptomatic cervical degenerative disc disease, however, it cause motion loss of the indexed level, increase the intradiscal pressure and motion of the adjacent levels, and may accelerate the degeneration of adjacent level. Cervical disc arthroplasty (CDA) was designed to preserve the motion of index level, avoid the over-activity of adjacent levels and reduce the degeneration of adjacent disc levels, the process of degeneration of adjacent level is very slowly, long term follow up studies should be conducted, this study aim to compare the more than 5 years’ long-term clinical outcomes and safety between CDA and ACDF.

**Methods:**

A systematic review and meta-analysis that will be performed according to the PRISMA (Preferred Reporting Items for Systematic Reviews and Meta-Analyses). The electric database of Medline, Embase, and Cochrane library will be systematic search. A standard data form will be used to extract the data of included studies. We will assess the studies according to the Cochrane Handbook for Systematic Reviews of Interventions, and perform analysis in software STATA 12.0. Fixed-effects models will be used for homogeneity data, while random-effects will be used for heterogeneity data. The overall effect sizes will be determined as weighted mean difference (WMD) for continuous outcomes and Relative risk (RR) for dichotomous outcomes.

**Results:**

The results of study will be disseminated via both international conference and peer-review journal.

**Conclusion:**

The conclusion of our study will provide the long-term and updated evidence of clinical outcomes and safety between CDA and ACDF, and help surgeon to change better surgical technique for patients.

## Abstract 2

Supplemental Digital Content is available in the text

**Strengths and limitations of this study**

1. Differ with that of previous publications, our study aims to compare more than 5-year clinical outcomes of cervical disc arthroplasty versus anterior cervical discectomy and fusion.
2. Our present study will provide useful evidence-based guidance for healthcare providers and policy stakeholder to facilitate the option of cervical disc arthroplasty for cervical degenerative diseases.
3. Our results may be limited by heterogeneity from different type dynamic devices.

## 1 Introduction

Cervical degenerative disc diseases are common in spinal disorders, and features with neck and arm pain, sometimes, associated with numbness of upper limbs, loss of function. The therapeutic methods include nonoperation, anterior cervical discectomy and fusion (ACDF), cervical disc arthroplasty (CDA), and others.^[1–3]^

Surgical intervention was recommended when the symptoms cannot be relieved by the nonoperative treatment.^[4,5]^ ACDF was almost the “golden standard” technique in treatment of symptomatic cervical degenerative disc disease, it need surgically remove the whole disc, and implant the cage or bone graft to achieve the aim of fusing the 2 adjacent vertebrae, many reports proved the good outcomes of this technique, which can significantly relieve the neck and arm pain, improve the function of patients.^[6–8]^

However, ACDF still had its drawbacks, it was reported the fused vertebrae will induce motion loss of the indexed level, increase the intradiscal pressure and motion of the adjacent levels, and accelerate the degeneration of adjacent level.^[9–13]^ Eck et al^[14]^ biomechanically tested 6 cadaveric cervical spine specimens and found that intradiscal pressure of upper adjacent and lower adjacent levels were increased by 73.2% and 45.3%, respectively, at flexion, as well as significantly increased adjacent segmental motion, similar results were reported by Park et al.^[15]^ A retrospective cohort study^[16]^ followed up 672 patients with average duration of 31 months, they found that a total of 101 (15%) patients underwent revision surgery, and 47.5% of revision surgery was caused by adjacent segment disease. Chung et al^[17]^ followed up 177 patients who underwent ACDF with at least 10 years (mean 16.2 years) and found that 92.1% patients were observed with radiographic adjacent segment pathologies, 19.2% patients had clinical adjacent segment pathologies.

To overcome these drawbacks of ACDF technique, preserve the motion of index level, avoid the overactivity of adjacent levels caused by ACDF, reduce the degeneration of adjacent disc levels, and then decrease the secondary surgical rate of adjacent disc levels, many different kinds of dynamic devices, such as ProDisc-C (Synthes Spine USA Products; LLC, West Chester, PA, USA), Prestige disc (Medtronic; Memphis, TN, USA), Bryan disc (Medtronic; Memphis, TN, USA), KineflexlC (SpinalMotion, Inc. Mountain View, CA, USA), Modic-C (LDR Medical, Austin, TX, USA), and PCM (NuVasive, Inc, San Diego, CA, USA), were designed and applied in clinical practice.^[18–25]^ However, Yin et al^[26]^ performed a meta-analysis including 13 reports from 10 randomized controlled trials (RCTs), the mean follow-ups of those 10 trials ranged from 1 to 5 years, they found that the operative rate at adjacent levels between CDA and ACDF was similar, with relative risk (RR) 95% confidence interval (CI) = 0.62 (0.31–1.27). Another meta-analysis^[27]^ focusing on adjacent segment degenerative and diseases also found no significant difference between CDA and ACDF. Because of the process of degeneration of adjacent level is very slow, long-term follow-up studies should be conducted to observe it.^[17]^ Recently, many authors reported the more than 5 years’ results of CDA versus ACDF,^[18,19,24,28–30]^ the more than 5 years’ long-term clinical efficacy and safety of dynamic devices in the cervical spine was still in debate.

Therefore, this study aims to compare more than 5 years’ long-term clinical outcomes and safety between CDA and ACDF and comprehensively evaluate the current long-term evidence to guide our clinical practice.

## 2 Methods and analysis

### 2.1 Phase I systematic review and identification of the included studies

#### 2.1.1 Study design

A systematic review and meta-analysis based on prospective RCTs with minimum 5-year follow-up was performed. This protocol was performed according to PRISMA-P (Preferred Reporting Items for Systematic review and Meta-Analysis Protocols) (Guidelines Checklist, Supplementary file).^[31]^

#### 2.1.2 Study registration

This systematic review and meta-analysis was registered with PROSPERO 2016 (no. CRD42016043155, Supplementary file). The study will be performed according to the PRISMA, and the Checklist PRISMA 2009 will be used to check our final reports.^[32,33]^

#### 2.1.3 Criteria of eligibility

##### 2.1.3.1 Study types

*Inclusion*. Only the prospective RCTs that compare the outcomes of CDA versus ACDF were considered, and the minimum follow-up must be more than 5 years.

*Exclusion*. RCTs follow-up less than 5 years, nonrandomized studies, case–control, case-cohort, observational studies, experimental studies, case series, and reviews will be excluded.

*Other criteria*. The RCTs must report complete efficacy data of ACDF and CDA treatment. Duplicated studies and studies that treated with ACDF and CDA hybrid technique will be excluded.

#### 2.1.4 Participants

Patients without limitation of age, gender, or ethnicity described as having cervical disc disease and need surgical intervention.

#### 2.1.5 Interventions

Any anterior artificial dynamic device that was used to perform the CDA will be included, such as ProDisc-C, Prestige disc, Bryan disc, KineflexlC, Modic-C, and PCM. The control group was treated by standard ACDF.

#### 2.1.6 Outcomes measures

##### 2.1.6.1 Primary outcomes

1. The pain of arm or neck will be assessed by VAS (visual analog scale) scores.
2. The function will be assessed by the NDI (neck disability index), SF-36 PCS (short form-36 physical component scores), neurological success, oversuccess, and work status.

##### 2.1.6.2 Secondary outcomes

1. Complications: including dural tear–, wound infection–, and implants-related complications such as device migration, subsidence, or failure.
2. Secondary surgery both in index and adjacent segments.

#### 2.1.7 Search methods and strategies

The electric database of Medline, Embase, and Cochrane library will be systematically searched without language restriction in July 2016 by 2 independent authors (MMS and CHC). The keywords will be used as follows: cervical disc arthroplasty, total cervical disc replacement, cervical dynamic device, cervical artificial disc, randomized controlled trial, randomized trial, and controlled clinical trial; the keywords will be combined with Boolean operators of AND, OR, and NOT. A search strategy developed with comprehensive use of keywords is shown in Table 1. The function of “related article” will also be used for search. The reference studies of previous systematic reviews, meta-analysis, and RCTs were manually searched to avoid initial miss.

**Table 1:**
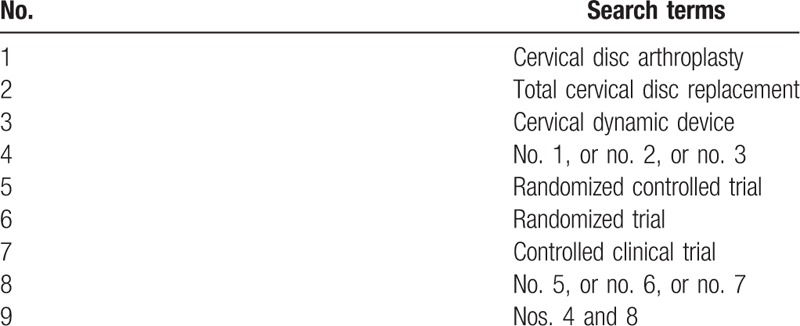
The developed search strategy for database of MEDLINE.

### 2.2 Phase II: study evaluation and data collection

#### 2.2.1 Selection of literature

The PRISMA flow diagram will be used for reporting these systematic reviews and meta-analysis (PRISMA 2009 flow diagram). The results of the literature search will be imported into software Endnote X4 (Clarivate Analytics, Philadelphia, PA, USA); 2 independent authors (XYW and ZKL) will screen the titles and abstracts to exclude studies that were duplicated or apparently irrelevant or clearly do not meet our inclusion criteria; the remaining studies will be download full text for reviewing and assessing the eligibility for inclusion. Any disagreements between the above 2 authors will be discussed and resolved with the third independent author (AMW).

#### 2.2.2 Data extraction

After confirming the included studies for systematic review and meta-analysis, 2 authors (MMS and CHC) will independently extract the data. A standard data extracted form, including general study characteristics (e.g., study design, the first author's name, the publish date, sample size of both groups, follow-up term, interventions, and controls); clinical outcomes (the VAS of both neck and arm pain, the NDI, the SF-36 PCS, neurological success, oversuccess, and work status); complications (dural tear–, wound infection–, and implant-related complications such as device migration, subsidence, or failure); and secondary surgery of index and adjacent levels. Quantitative data will be extracted to calculate effect sizes. For continuous outcomes, the mean and standard deviation will be extracted, for dichotomous outcomes, the numbers of events in both ACDF and CDA group will be extracted. The data in other forms will be recalculated to enable pooled analysis, when possible. Two other authors will review the data to confirm the accuracy of the extracted data. All of the extracted data will be input and briefly summarized (Table 2). We will contact the author to obtain the missing data.

**Table 2:**
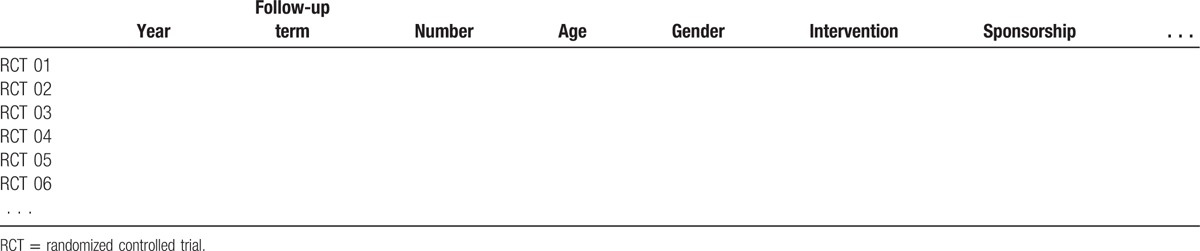
The summary data of all included RCTs.

#### 2.2.3 Risk of bias assessment

The risk of bias of the included studies will be assessed according to the Cochrane Handbook for Systematic Reviews of Interventions, which includes 7 domains: random sequence generation, allocation concealment, blinding of participants and personnel, blinding of outcome assessment, incomplete outcome data addressed, selective reporting, and other biases. In addition, the judgments of reviewers are classified as “low risk,” “high risk,” or “unclear risk” of bias (Table 3). The overall quality of this systematic review and meta-analysis will be summarized and evaluated with GRADEpro (http://www.gradepro.org).

**Table 3:**
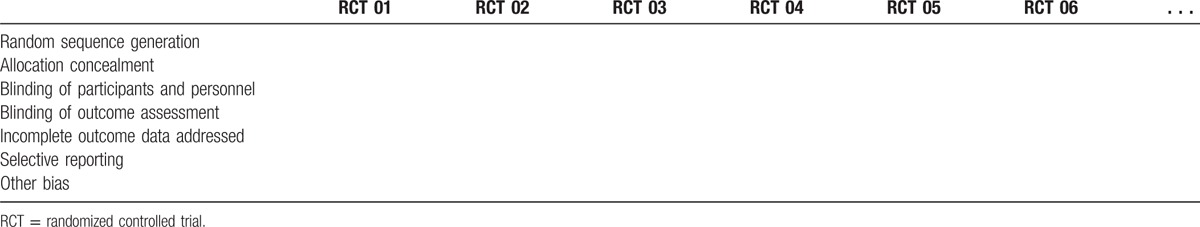
Risk of bias assessment of all included studies.

### 2.3 Phase III: statistical analysis

#### 2.3.1 Data synthesis

The meta-analysis will be performed with the statistic software STATA 12.0 (StataCorp, College Station, TX). Fixed-effects models or random-effects models will be chosen according to the heterogeneity of the included studies, fixed-effects models will be used for homogeneous data (*I*^2^ < 50%), while random-effects will be used for heterogeneous data (*I*^2^ > 50%). The overall effect sizes will be determined as weighted mean difference for continuous outcomes and RR for dichotomous outcomes with 95% CIs.

#### 2.3.2 Heterogeneity

*X*^2^ and *I*^2^ tests will be used to evaluate the heterogeneity of included studies. We define the acceptable heterogeneity if *I*^2^ < 50% and *P* > 0.10 (*X*^2^ test), and significant heterogeneity if *I*^2^ > 50% or *P* < 0.10.^[34]^ To determine the possible cofounders affecting the outcomes, meta-regression and subgroup meta-analysis will be conducted.

If the included studies from different countries (most subgroup divided by different countries have more than included 2 studies), using different type of cervical artificial disc (most subgroup divided by different type of cervical artificial disc have more than included 2 studies), some others factors such as age, gender, and race. Subgroup analysis of these factors will be conducted.

Sensitivity analysis will also be performed to omit the potential inconsistent study. If removing 1 study will affect the other results markedly, this inconsistent study will be removed, and the results of the remaining studies will be conducted by fixed-effects models.

#### 2.3.3 Publication bias

Both Egger et al^[35]^ and Begg and Mazumdar^[36]^ funnel plot will be performed to assess the publication bias, and we will use the contour-enhanced funnel plot to distinguish asymmetry that may be caused by publication bias or other factors.

#### 2.3.4 Ethical issues

No primary personal data will be collected, and no additional ethical approval needs to be obtained.

#### 2.3.5 Publication plan

The protocol of this meta-analysis was registered with PROSPERO (International Prospective Register of Systematic Reviews), with registration number—CRD42016043155. The results of present meta-analysis will be submitted to international conference peer-reviewed journal, after being accepted and published on journal, the raw data of present study will be freely available online.

## 3 Discussion

The dynamic artificial cervical designed for the purpose to avoid the overactivity of adjacent levels, therefore, may slowdown the degeneration of adjacent disc levels and decrease the rate of secondary surgery. However, the cervical disc degeneration is a slow process, and the primary or mid-term (less than 5 years) results were followed up too short. The long term results that more than 5 years were the surgeons concern.

We found that many authors reported more than 5 years’ long-term results of CDA versus ACDF,^[18,19,24,28–30]^ and still no one conducted a meta analysis based on RCTs with the minimized 5-year clinical outcome. Therefore, our present study will give surgeons a long-term evidence-based results on whether the CDA can decrease the degeneration and secondary surgery of adjacent disc levels; meanwhile, we will also answer the question whether the CDA maintains its pain relief and functional improvement at long-term time point.

Summary, our present protocol will compare both the long-term efficacy and safety of CDA versus ACDF in treatment of degenerative cervical disease; the results will be disseminated through both international conference and peer-review journal.

## Supplementary Material

Supplemental Digital Content
