# Comparison of the more than 5-year clinical outcomes of cervical disc arthroplasty versus anterior cervical discectomy and fusion: A protocol for a systematic review and meta-analysis of prospective randomized controlled trials: Erratum

**DOI:** 10.1097/MD.0000000000006219

**Published:** 2017-02-24

In the article “Comparison of the more than 5-year clinical outcomes of cervical disc arthroplasty versus anterior cervical discectomy and fusion: A protocol for a systematic review and meta-analysis of prospective randomized controlled trials”,^[1]^ which appeared in Volume 95, Issue 51 of *Medicine*, affiliations were listed incorrectly. Chun-Hui Chen, Zhong-Ke Lin, Xiang-Yang Wang, Qi-Shan Huang, Yong-Long Chi, and Ai-Min Wu are solely affiliated with the Department of Orthopedics, Second Affiliated Hospital of Wenzhou Medical University, Second Medical College of Wenzhou Medical University, Wenzhou, Zhejiang, People's Republic of China.
