# Supplementary material for: Comparison of the more than 5-year clinical outcomes of cervical disc arthroplasty versus anterior cervical discectomy and fusion: A protocol for a systematic review and meta-analysis of prospective randomized controlled trials
Source: Medicine (Baltimore). 2016 Dec 23;95(51):e5733. doi: 10.1097/MD.0000000000005733 (PMC5181829; doi:10.1097/MD.0000000000005733)
Supplement: Supplemental Digital Content [file medi-95-e5733-s001.pdf]

Comparison of the more than five-year clinical outcomes of cervical disc arthroplasty versus anterior cervical discectomy and fusion: a protocol for a systematic review and meta-analysis of prospective randomized controlled trials

Ai-Min Wu

Citation

Ai-Min Wu. Comparison of the more than five-year clinical outcomes of cervical disc arthroplasty versus anterior cervical discectomy and fusion: a protocol for a systematic review and meta-analysis of prospective randomized controlled trials. PROSPERO 2016:CRD42016043155 Available from
http://www.crd.york.ac.uk/PROSPERO_REBRANDING/display_record.asp?ID=CRD42016043155

Review question(s)

What is the more than five-year long-term clinical outcomes of cervical disc arthroplasty versus anterior cervical discectomy and fusion.

Searches

The electronic database of MEDLINE, EMBASE, and Cochrane library will be systematic search without language restriction at July 2016 by two independent authors.

Types of study to be included

Only the prospective randomized controlled trials that compare the outcomes of cervical disc arthroplasty versus anterior cervical discectomy and fusion were considered, and the minimum follow up must be more than 5 years.

Condition or domain being studied

Cervical degenerative disc diseases are common in spinal disorders, and features with neck and arm pain, sometimes, associated with numbness of upper limbs, loss of function. anterior cervical discectomy and fusion was almost the “golden standard” technique in treatment of symptomatic cervical degenerative disc disease, However, ACDF still had its drawbacks, it was reported the fused vertebrae will induce motion loss of the indexed level, increase the intradiscal pressure and motion of the adjacent levels, and accelerate the degeneration of adjacent level. To overcome these drawbacks of ACDF technique, preserve the motion of index level, avoid the over-activity of adjacent levels caused by ACDF, reduce the degeneration of adjacent disc levels, and then decrease the secondary surgical rate of adjacent disc levels, Many different kinds of dynamic devices, such as ProDisc-C, Prestige disc, Bryan disc, KineflexlC , Modic-C and PCM, were designed and applied in clinical practice.

Participants/ population

Patients without limitation of age, gender or ethnicity described as having cervical disc disease, and need surgical intervention.

Intervention(s), exposure(s)

Any anterior artificial dynamic device was used to perform the cervical disc arthroplasty will be included, such as ProDisc-C, Prestige disc, Bryan disc, KineflexlC , Modic-C and PCM.

Comparator(s)/ control

The control group was treated by standard anterior cervical discectomy and fusion.

Outcome(s)

Primary outcomes

1. The pain of arm or neck will be assessed by VAS (Visual analogue scale) scores.

2. The function will be assessed by the NDI (Neck disability index), SF-36 PCS (Physical component scores),

neurological success, over success and work status.

Secondary outcomes

1. Complications: including dural tear, wound infection, and implants related complication such as device migration, subsidence, or failure.

2. Secondary surgery both in index and adjacent segments.

Data extraction, (selection and coding)

Two authors will independently extracted the data. A standard data extracted form, including:

- general study characteristics (eg: study design, the first author's name, the publish date, sample size of both groups, follow-up term, interventions and controls);

- clinical outcomes (The VAS of both neck and arm pain, the NDI (Neck disability index), The SF-36 PCS (Physical component scores), neurological success, over success and work status);

- complications (Dural tear, wound infection, and implants related complication such as device migration, subsidence, or failure);

- secondary surgery of index and adjacent levels.

Risk of bias (quality) assessment

The risk of bias of the included studies will be assessed according to the Cochrane Handbook for Systematic Reviews of Interventions, which includes seven domains:

- random sequence generation;

- allocation concealment;

- blinding of participants and personnel;

- blinding of outcome assessment;

- incomplete outcome data addressed;

- selective reporting;

- other bias.

And the judgments of reviewers are classified as "low risk," "high risk," or "unclear risk" of bias.

Strategy for data synthesis

The meta-analysis will be performed with the statistic software STATA 12.0 (StataCorp, College Station, TX). Fixed-effects models or random-effects models will be chosen according to the heterogeneity of included studies, fixed-effects models will be used for homogeneity data (I2 <50%), while random-effects will be used for heterogeneity data (I-squared >50%). The overall effect sizes will be determined as weighted mean difference (WMD) for continuous outcomes and Relative risk (RR) for dichotomous outcomes with 95% confidence intervals (CIs).

Analysis of subgroups or subsets

If the included studies from different countries (most subgroup divided by different countries have more than included 2 studies), using different type of cervical artificial disc (most subgroup divided by different type of cervical artificial disc have more than included 2 studies), some others factors such as age, gender, and race. Subgroup analysis of these factors will be conducted.

Dissemination plans

The results of present meta-analysis will be submitted to international conference peer-reviewed journal, after accepted and published on journal, the raw data of present study will be free available online.

Contact details for further information

Dr Wu

109# Xueyuan Xi Road

aiminwu2005@163.com

Organisational affiliation of the review

None

Review team

Dr Ai-Min Wu,

Collaborators

Dr Min-Min Shao, Wenzhou Center Hospital, Dingli Hospital of Wenzhou Medical University
Dr Chun-Hui Chen, Second Affiliated Hospital of Wenzhou Medical University, Second Medical College of Wenzhou Medical University
Dr Zhong-Ke Lin, Second Affiliated Hospital of Wenzhou Medical University, Second Medical College of Wenzhou Medical University
Dr ,

Anticipated or actual start date

01 July 2016

Anticipated completion date

30 September 2016

Funding sources/sponsors

This work was funded by the National Natural Science Foundation of China (81501933, 81372014), Wenzhou Science and Technology Project (2016Y0469), and Natural Science Foundation of Zhejiang Province (LY14H060008).

Conflicts of interest

None known

Language

English

Country

China

Subject index terms status

Subject indexing assigned by CRD

Subject index terms

Arthroplasty; Cervical Vertebrae; Diskectomy; Humans; Intervertebral Disc; Prospective Studies; Randomized Controlled Trials as Topic

Stage of review

Ongoing

Date of registration in PROSPERO

19 July 2016

Date of publication of this revision
31 August 2016

**Stage of review at time of this submission**	**Started**	**Completed**
Preliminary searches	Yes	No
Piloting of the study selection process	No	No
Formal screening of search results against eligibility criteria	No	No
Data extraction	No	No
Risk of bias (quality) assessment	No	No
Data analysis	No	No

PROSPERO
International prospective register of systematic reviews
The information in this record has been provided by the named contact for this review. CRD has accepted this information in good faith and registered the review in PROSPERO. CRD bears no responsibility or liability for the content of this registration record, any associated files or external websites.
